# An Influence of Birth Weight, Gestational Age, and Apgar Score on Pattern Visual Evoked Potentials in Children with History of Prematurity

**DOI:** 10.1155/2015/754864

**Published:** 2015-08-31

**Authors:** Marta Michalczuk, Beata Urban, Beata Chrzanowska-Grenda, Monika Oziębło-Kupczyk, Alina Bakunowicz-Łazarczyk

**Affiliations:** Department of Pediatric Ophthalmology and Strabismus, Medical University of Bialystok, Waszyngtona 17, 15-274 Bialystok, Poland

## Abstract

*Purpose*. The objective of our study was to examine a possible influence of gestational age, birth weight, and Apgar score on amplitudes and latencies of P100 wave in preterm born school-age children. *Materials and Methods*. We examined the following group of school-age children: 28 with history of prematurity (mean age 10.56 ± 1.66 years) and 25 born at term (mean age 11.2 ± 1.94 years). The monocular PVEP was performed in all children. *Results*. The P100 wave amplitudes and latencies significantly differ between preterm born school-age children and those born at term. There was an essential positive linear correlation of the P100 wave amplitudes with birth weight, gestational age, and Apgar score. There were the negative linear correlations of P100 latencies in 15-minute stimulation from O1 and Oz electrode with Apgar score and O1 and O2 electrode with gestational age. *Conclusions*. PVEP responses vary in preterm born children in comparison to term. Low birth weight, early gestational age, and poor baseline output seem to be the predicting factors for the developmental rate of a brain function in children with history of prematurity. Further investigations are necessary to determine perinatal factors that can affect the modified visual system function in preterm born children.

## 1. Introduction

A premature birth of a child entails an insufficient formation of multiple organ structures that might result in their dysfunction [1, 2]. It is known that premature infants exhibit neurodevelopmental delay and reduced growth of the cerebral cortex. Malik et al. showed that glutamatergic neurogenesis continues in the premature infants, and preterm birth suppresses neurogenesis [3]. They suggest hypoxia-mimetic agents might restore neurogenesis, enhance cortical growth, and improve neurodevelopmental outcome of premature infants. Children born preterm are at risk of visual impairment due to cerebral visual impairment, which is caused by damage to the geniculocalcarine pathways and is related to the severity of white matter injury [4]. In preterm infants, the periventricular white matter is particularly susceptible to injury. White matter contains important subcortical pathways including the corticospinal tracts and optic radiations. The visual function deficits seen in children born prematurely may be related to the networks involving the cortical dorsal stream and its connections to the parietal, frontal, and hippocampal areas [5]. On the other hand, despite the immaturity of the visual pathway in preterm infants, Jandó et al. showed that the visual cortex is remarkably ready to accept environmental stimulation right after birth [6]. This early plasticity makes full use of the available extra stimulation time in preterm human infants and results, for example, in an early onset of cortical binocularity [7].

Frequently, children born small for gestational age exhibit poor initial general condition, including onset of a retinopathy of prematurity [1, 2]. Likewise, the microstructure of the central nervous system diverges in neonates with a gestational age less than 38 weeks from children born at term [8]. Importantly, the formation of the central nervous system does not end in the moment of a birth but persists throughout childhood [9, 10]. Proper development of the central nervous system is determined by morphological growth of the visual cortex until 11 years of age and its metabolic formation until 18 years of age [4, 5]. Extensive researches have been conducted on the visual system function by means of pattern visual evoked potentials (PVEPs), revealing that PVEP responses change in time [11, 12]. Children demonstrate higher latencies and higher amplitudes when compared to adults [13, 14]. However, electrophysiological activity matures [12, 15]. Throughout childhood, decrease in latencies and decrease in amplitudes of P100 wave can be observed [10, 11]. Furthermore, P100 wave presents altered range of values in children with history of prematurity in comparison to those born at term [11]. It has been reported that age-dependent PVEP alterations in preterm born neonates seem to remain in close connection to the structural changes in the visual cortex [15]. Moreover, an anomalous primary conformation of the central nervous system implies its delayed path of development, which might be reflected in modified results of pattern visual evoked potentials [12, 16].

Although changed maturation of the electrophysiological responses caused by prematurity is highly proven, there is lack of objective data if gestational age, birth weight, and Apgar score influence P100 amplitudes and latencies. The present study used pattern visual evoked potentials to examine the function of the visual system in preterm born school-age children. We hypothesized that if the actual outcomes are associated with the history of prematurity, higher latencies and lower amplitudes of P100 wave should be observed in the study group, in comparison to peers born appropriate for gestational age. Furthermore, we wanted to determine whether PVEP parameters correlate with birth weight, gestational age, and Apgar scores.

## 2. Materials and Methods

The current study was performed at the Department of Pediatric Ophthalmology and Strabismus, Medical University of Bialystok, Poland. The study and its testing procedures were approved by the University Ethic Committee and were in accordance with the Declaration of Helsinki.

We examined 28 school-age children with a history of prematurity. The results were compared to controls. Inclusion criteria to the study group were as follows: best corrected visual acuity on 1.0 level and preterm birth. Exclusion criteria were high myopia, optic nerve pathology, any disease affecting the central nervous system, and changes in neonatal transfontanel ultrasonography. For final analysis, 50 eyes were involved, while 2 eyes of 2 patients were excluded because of glaucoma and 1 eye of 2 patients was excluded due to high myopia in one eye. The retina of 18 eyes was treated with laser in infancy.

Twenty-five school-age children born at term were enrolled in the control group (mean age 11.2 ± 1.94 years). Boys make up a 36 and girls a 64 percentage of the test group. No history of diseases affecting optic nerve, retina, or central nervous system was found. Visual acuity was equal to 1.0 in all subjects. PVEPs of those children served as the norms by the electrophysiology lab in the past 5 years.

The PVEP examination was performed in a lab with accordance to ISCEV standards by the use of the Espion Diagnosys equipment. PVEP responses were recorded by 5 gold-plated cup electrodes which were attached according to the 10–20 System of Electrode Placement. Three active electrodes were placed on the occipital scalp (O1: left hemisphere, Oz: midline, and O2: right hemisphere). The ground electrode was located on the vertex. The reference electrode was set on Fz. The patients were seated one meter viewing distance from the monitor (AccuSync 120) on which a black and white checkerboard was displayed. A used black and white checkerboard pattern was 15 and 60 minutes of arc. All checks were square and the number of light and dark squares was equal. A red fixation point was positioned at a corner of four checks which were located at the center of the field. The mean luminance of the checkerboard was 100 cd/m and the contrast between black and white squares was equal to 100%. Reversal rate was equal to 1.999 per sec. Sweeps per result were 80. Low cut-off filter was 2.5 Hz and high cut-off 100 Hz. The average response was obtained from 2 reversals which were adequate in P100 amplitude and latency. The P100 wave was measured from the preceding N75 peak. The monocular stimulation was performed.

### 2.1. Statistical Analysis

Statistic data was processed using STATISTICA Version 10 (StatSoft). The Kolmogorov-Smirnov test (KS-test), chi-square distribution, Student's *t*-test, scatter diagrams, and Pearson's correlation coefficient (*r*) were processed for statistical analysis. KS-test was performed to delimit normality of the distribution. Chi-square distribution was executed to confirm that there are no statistically important differences in percentage of boys and girls between test and control group. Student's *t*-test was accomplished to determine possible age differences between the test and the control group. The P100 wave latencies and amplitudes in 15- and 60-minute pattern stimulation obtained from O1, Oz, and O2 electrode were compared for analogical age in test and control group (Student's *t*-test). Afterwards, correlations were determined between the P100 wave latencies, amplitudes and gestational age, birth weight, and Apgar score. PCC was computed to determine dependencies between variables. Scatter diagrams were prepared to visualize correlations between PVEP parameters gestational age, birth weight, and Apgar score. Differences with a *P* value less than 0.05 were considered statistically significant.

## 3. Results

Mean age in the study group was 10.56 ± 1.66 years. Boys make up a 52 and girls a 48 percentage of the test group. Mean gestational age was 30 ± 3.54 weeks. Mean birth weight was 1524.4 ± 580.96 grams. Mean Apgar score in 5 minutes was 5.24 ± 2.8.

KS-test confirmed that data was distributed normally. Chi-square distribution showed no statistically important differences in percentage of boys and girls between test and control group. Student's *t*-test revealed no statistically important age differences between the test and the control group.

### 3.1. P100 Latencies

The latencies of P100 wave in 15- and 60-minute check stimulation vary in school children with history of prematurity in comparison to their peers (Student's *t*-test). The P100 latency was delayed in the study group in comparison to controls (Table 1).

**Figure 1:**
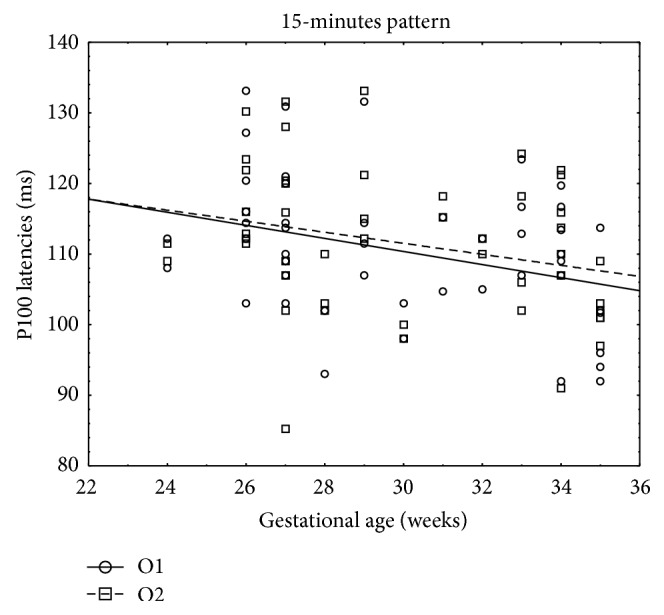
Scatter diagram showing a negative linear correlation between P100 latencies (ms) and birth weight (grams) in 15-minute pattern stimuli.

**Figure 2:**
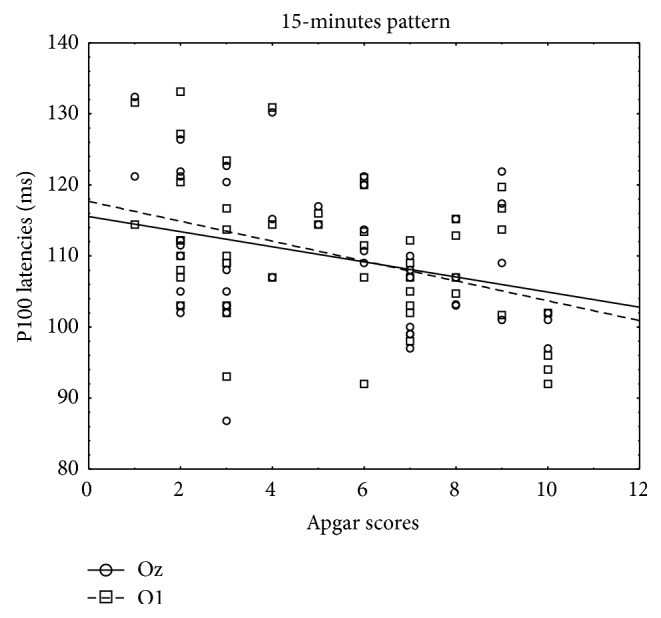
Scatter diagram showing a negative linear correlation between P100 latencies (ms) and amount of Apgar score in 15-minute pattern stimuli.

Pearson's correlation coefficient was determined to evaluate the relationship of the P100 wave latencies obtained from O1, Oz, and O2 electrode with gestational age, birth weight, and Apgar score. There was a negative correlation between the P100 latency in 15-minute check stimuli and gestational age, birth weight, and Apgar score. Also P100 latencies in 60-minute check stimuli correlated negatively with gestational age, birth weight, and Apgar score. To conclude, there was a negative relationship between P100 latencies and gestational age, birth weight, and Apgar score. Higher results of P100 wave latencies were correlated with earlier gestational age, smaller birth weight, and lower Apgar score. However, there were only correlations in 15-minute check stimuli with amount of Apgar score (obtained from O1 and Oz electrode) and gestational age (obtained from O1 and O2 electrode) (Table 2). Scatter diagrams highlight the essential correlations (Figures 1 and 2).

### 3.2. P100 Amplitudes

**Figure 3:**
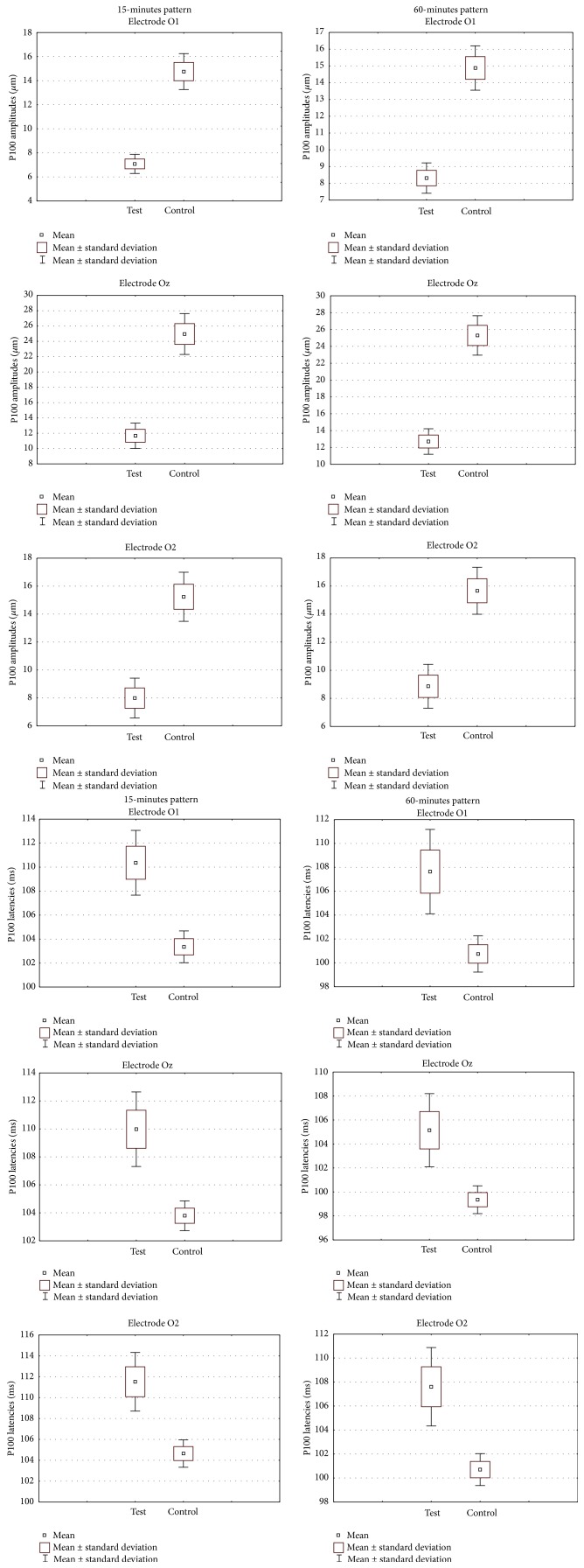
Whiskers diagrams highlight P100 wave amplitudes (*μ*m) and P100 wave latencies (ms) significant differences between the test and the control group.

The P100 wave amplitudes differ between preterm born school-age children and those born appropriate for gestational age (Student's *t*-test). The P100 amplitudes were smaller in the study group in comparison to the control group: from O1 electrode (in *μ*m): 7.08 ± 2.87 versus 14.75 ± 5.38 in 15 min. (*P* < 0.001) and 8.31 ± 3.27 versus 14.88 ± 4.77 in 60 min. (*P* < 0.001); from Oz electrode: 11.67 ± 5.98 versus 24.96 ± 9.62 in 15 min. (*P* < 0.001) and 12.71 ± 5.46 versus 25.3 ± 8.44 in 60 min. (*P* < 0.001); from O2 electrode: 7.99 ± 5.14 versus 15.24 ± 6.33 in 15 min. (*P* < 0.001) and 8.86 ± 5.63 versus 15.67 ± 6.04 in 60 min. (*P* < 0.001) (Figure 3).

**Figure 4:**
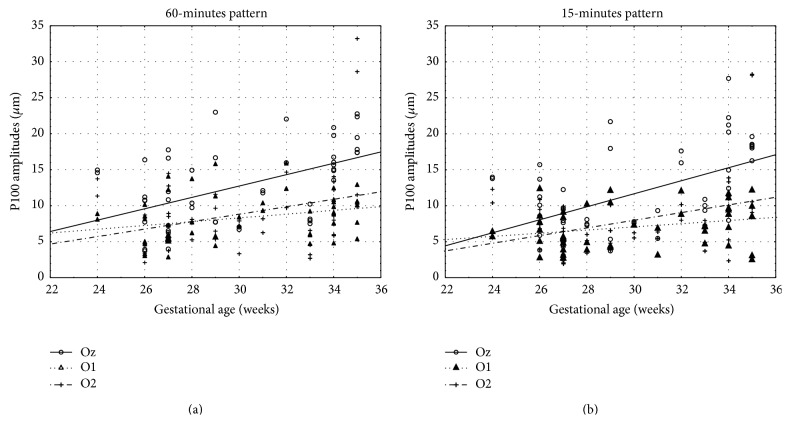
Scatter diagram showing a positive linear correlation between P100 amplitudes (*μ*m) and gestational age (weeks) in 15- and 60-minute pattern stimuli.

**Figure 5:**
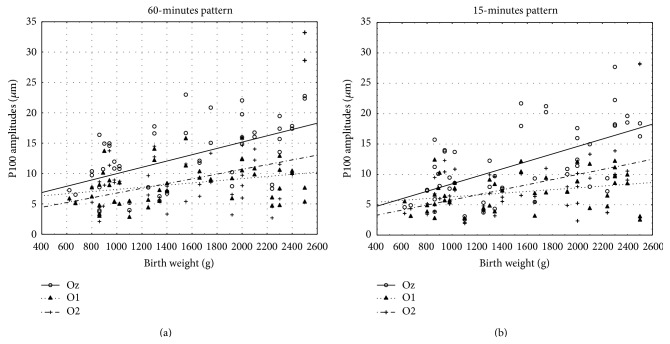
Scatter diagram showing a positive linear correlation between P100 amplitudes (*μ*m) and birth weight (grams) in 15- and 60-minute pattern stimuli.

**Figure 6:**
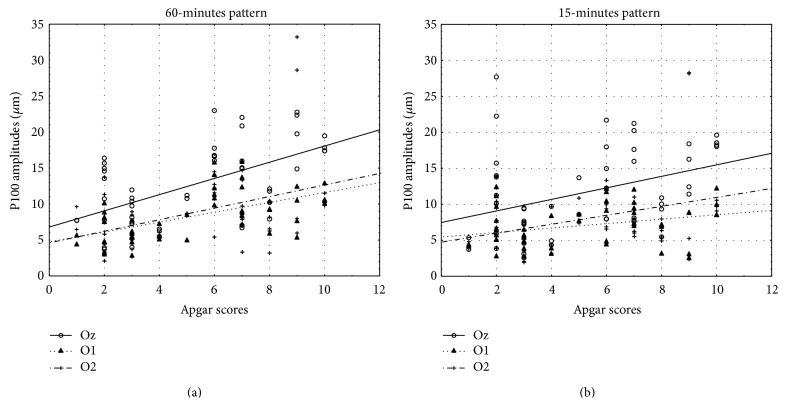
Scatter diagram showing a positive linear correlation between P100 amplitudes (*μ*m) and amount of Apgar score in 15- and 60-minute pattern stimuli.

To assess the correspondence of the P100 wave amplitude values in 15- and 60-minute pattern stimulation (obtained from O1, Oz, and O2 electrode) with gestational age, birth weight, and Apgar score, a PCC was gauged. There was a positive correlation between P100 amplitudes in 15-minute check stimulation and gestational age, birth weight, and amount of Apgar score. Also the values of P100 amplitudes in 60-minute pattern stimulation correlated positively with gestational age, birth weight, and amount of Apgar score. Overall, there was a positive correlation of the P100 amplitudes with gestational age, birth weight, and Apgar score. Higher P100 wave amplitudes values were correlated with later gestational age, greater birth weight, and higher amount of Apgar score (Table 2). Scatter diagrams highlight the correlations (Figures 4, 5, and 6).

## 4. Discussion

Brain development in the late preterm period is essential for proper cognitive abilities [8, 17]. It is only 38–40 weeks after conception that elongation of dendrites and proper dendritic branching is completed [18, 19]. The influence of the preterm birth and low gestational weight on the global and regional brain volume abnormalities was proven by many authors [8, 16, 20]. Moreover, Peterson et al. have demonstrated that these brain volume differences between term and preterm born children persisted until later childhood [20]. Peterson et al. and Ball et al. described the association of lower mean diffusivity in occipital lobes with preterm birth [8, 20]. MRI investigations performed by Parikh et al. revealed decreased regional and total brain tissue volume in extremely low birth infants [16]. It is known that oxygen contributes to the pathogenesis of neonatal brain damage, leading to neurocognitive impairment of prematurely born infants in later life. Felderhoff-Mueser reported that short exposures to nonphysiologic oxygen levels cause oxidative stress and can trigger apoptotic neurodegeneration in the developing brains of infant rodents [21]. Sifringer et al. reported that hyperoxia triggers a marked increase in the active caspase-2 expression, resulting in an initiation of the intrinsic apoptotic pathway with upregulation of key proteins [22].

Maturation of the visual system is still thoroughly studied by neurologists and ophthalmologists. Many researchers have investigated global and microstructural brain tissue changes in the development of the central nervous system and age-dependent alterations of visual evoked potentials responses [8–12, 15, 16, 18–20]. Also the evolutional differences between prematurely born children and those born appropriate for gestational age are still substantial [11, 16, 23–25]. However, the main current and future task for neurological and ophthalmological research is the exploration of the factors that can affect the modified visual system function in preterm born subjects [23].

The structural changes in the central nervous system seem to be reflected in the PVEPs alterations [15]. In our study we observed that there was an essential positive linear correlation of the P100 wave amplitudes with birth weight, gestational age, and Apgar score. We also noticed the negative linear correlation of P100 latencies in 15 minutes simulation with Apgar score and gestational age. Sokol and Jones explored that children born preterm had shorter latencies of P100 wave than children born appropriate for gestational age [11]. Inversely, Ruberto et al. revealed that premature newborns had delayed latencies of P100 wave in comparison to neonates born at term [24]. Although Peterson et al.'s research on central nervous system development confirmed that structural brain changes persisted until later childhood, Nilsson et al. ascertained that children born small for gestational age show no signs of accelerated neurophysiological maturation in preschool period [20, 25]. Moreover, Atkinson et al. demonstrated that visual development of children with only the history of prematurity is unchanged even in the neonatal period. However, the authors highlighted that there is a possible influence of perinatal damage factors which were reflected in modified PVEP responses [23].

The outcomes, which we achieved in our study, appear to be consistent with previous reports [9, 10, 15, 16, 20]. The P100 wave latencies were delayed and amplitudes were statistically smaller in the test group in comparison to controls. Increasing amplitudes and decreasing latencies of P100 wave in school-age children with history of prematurity are similar to Brecelj et al.'s results, obtained in the study on maturation of the electrophysiological responses [12, 15]. Similarly, our outcomes also seem to correspond with results obtained in research on structural development of the central nervous system described by Garey and de Courten and metabolic formation of the brain described by Huttenlocher et al. [9, 10]. Lower P100 amplitudes in the test group and the correlation between P100 amplitudes and birth weight, gestational age, and Apgar score might reflect a decrease in the total brain tissue volume in preterm born children described by Parikh et al. [16]. Moreover, Peterson et al.'s studies on the persistence of brain changes in children with a history of prematurity until childhood seem to confirm that correspondence [20]. The correlations between P100 latencies (15-minute check stimuli) and Apgar score or low birth weight demonstrate that initial general condition of a preterm born child has a significant influence on electrophysiological responses. That might confirm Atkinson's theory that there are perinatal damage factors that change PVEP responses [23].

Also the researches on flash visual evoked potentials (FVEPs) seem to correspond with our study on PVEP values. Giapros et al. concluded that FVEP developmental pattern of preterm infants was similar to that of healthy full-term infants; the former had deficits in visual electrophysiologic maturation, especially for very low birth weight children [26]. Feng et al. noticed that latency of the P2 main wave on FVEPs was delayed more significantly in premature infants than in full-term infants [27]. They ascertained that the visual functional development was delayed in preterm born infants, especially in infants with very low birth weight and in gestational age less than 32 weeks [27, 28]. However, FVEP is less precise examination than PVEP; thereby PVEP should be the first ordered testing [29].

In our study we examined preterm born school-age children, with mean age of 10.56 ± 1.66 years. There are only few publications concerning the analysis of the PVEP in a similar age group. Feng et al. evaluated PVEP in 61 preterm preschoolers with average intelligence quotients and compared them to 41 normal children [30]. The PVEP P100 wave latencies were significantly prolonged in the very low birth weight group compared with the controls, while showing delay in the low birth weight group. They concluded that preterm preschoolers with an average cognition capability are at the risk of defect in visual-spatial perception. In their opinion PVEP may provide an objective and convenient measurement in detecting the problem of visual perception in children. O'Reilly et al. examined 12 preterm born children and 12 born full-term controls at 8–12 years of age [31]. On the contrary, they observed that the P100 component of the PVEP showed a significantly shorter latency in the preterm compared with the full-term participants. Ruberto et al. tried to identify subclinical morphologic or functional defects in premature infants born between 28 and 35 weeks [7]. They evaluated PVEP, OCT, and HRT in 14 premature newborns at birth and subsequently when they were young children (mean age 7.5 ± 0.2 years). Multiple significant *P* values were found in the VEP P100 peak time and steady-state amplitudes at the time of birth, but not at the time of the morphologic analysis. They also observed statistically significant changes of the optic nerve in OCT and HRT. They concluded that healthy, premature newborns may have morphologic abnormalities of the optic nerve and these abnormalities do not cause visual acuity or functional decreases.

Our study confirmed that PVEP responses differed between preterm born school-age children and children born at term. However, we did not examine if there are changes in P100 parameters during school-age period, because we believe that the maturation of pattern visual evoked potentials is highly proven. The aim of our study was to objectivize the current knowledge about the influence of prematurity on pattern visual evoked potentials parameters. We believe that strong correlations between low birth weight, early gestational age, Apgar score, and P100 amplitudes or some P100 latencies seem to be the evidence that premature birth impacts effects on visual evoked responses. Furthermore, our research proves that this influence persists even until school-age. Positive linear correlations between P100 amplitudes and gestational age, Apgar score, and birth weight prove without a doubt their role as predicting factors for the developmental rate of a brain function in children with a history of prematurity. The negative linear correlations of P100 latencies in 15 minutes stimulation from O1 and Oz electrode with Apgar score, and from O1 and O2 electrode with gestational age, might reflect the delayed electrophysiological maturation for small pattern in comparison to the big one [32]. Nevertheless, further similar researches in a group of younger children that we examined had to be performed to validate this hypothesis.

Still, we acknowledge that our study has some limitations. Firstly, the number of patients in the control group and the study group was rather small. Secondly, the small amount of results prevents us from subdividing measurements. Therefore our study mixes low, very low, and extremely low birth weights. Also boys and girls groups are not assigned. The difference in the number of patients in the mentioned subgroups renders receiving credible and statistically important results impossible; the normality of data distribution cannot be achieved.

The data of PVEP responses in school-age children with history of prematurity is still lacking. Commonly, preterm born children with good visual acuity are not remaining under the supervision of an ophthalmologist. Further investigations are necessary to determine the perinatal factors that can affect the modified visual system function in preterm born persons. The current neonatal knowledge enables physicians to save lives of children with increasingly lower gestational age and birth weight. A matter of special interest will be the influence of low, very low, and extremely low birth weight and very small gestational age on PVEP responses. However, also different perinatal factors, especially changes in perinatal care over the past 30 years, will be the issue of increasing importance.

## 5. Conclusions

PVEP responses vary in preterm born children in comparison to their peers born appropriate for gestational age. Low birth weight, early gestational age, and poor baseline output seem to be the predicting factors for the developmental rate of a brain function in children with history of prematurity. Further investigations are necessary to determine perinatal factors that can affect the modified visual system function in preterm born persons.

## Figures and Tables

**Table 1:** PVEP values in the test group and the control group (Student's *t*-test, *n*: number of eyes).

PVEP electrode	PVEP parameters	Control group (*n* = 50) Mean ± SD		Test group (*n* = 50) Mean ± SD		Significance	
		15 min	60 min	15 min	60 min	15 min	60 min
O1	P100 latency (ms)	103.36 ± 4.77	100.74 ± 5.48	110.37 ± 9.74	107.64 ± 12.74	*P* < 0.001^*∗*^	*P* < 0.001^*∗*^
Oz	P100 latency (ms)	103.79 ± 3.84	99.35 ± 4.19	109.97 ± 9.61	105.15 ± 11.02	*P* < 0.001^*∗*^	*P* < 0.001^*∗*^
O2	P100 latency (ms)	104.65 ± 4.73	100.7 ± 4.81	111.52 ± 10.13	107.61 ± 11.79	*P* < 0.001^*∗*^	*P* < 0.001^*∗*^

^*∗*^Statistically significant.

**Table 2:** Correlation of PVEP variables with gestational age (weeks), birth weight (grams), and Apgar score.

PVEP electrode	PVEP parameters (*n* = 50)		GA (weeks)		Birth weight (grams)		Apgar score	
			15 min	60 min	15 min	60 min	15 min	60 min
O1	P100 latency (ms)	*r*	−0.34^*∗*^	−0.04	−0.23	−0.02	−0.4^*∗*^	−0.14
	P100 amplitude (*µ*m)	*r*	0.27^*∗*^	0.29^*∗*^	0.3^*∗*^	0.31^*∗*^	0.3^*∗*^	0.58^*∗*^

Oz	P100 latency (ms)	*r*	−0.21	−0.13	−0.10	−0.1	−0.3^*∗*^	−0.18
	P100 amplitude (*µ*m)	*r*	0.53^*∗*^	0.50^*∗*^	0.59^*∗*^	0.55^*∗*^	0.37^*∗*^	0.58^*∗*^

O2	P100 latency (ms)	*r*	−0.27^*∗*^	−0.15	−0.19	−0.15	−0.26	−0.16
	P100 amplitude (*µ*m)	*r*	0.37^*∗*^	0.33^*∗*^	0.47^*∗*^	0.40^*∗*^	0.34^*∗*^	0.4^*∗*^

GA: gestational age; *n*: number of eyes; *r*: Pearson's correlation coefficient; ^*∗*^correlation.
